# Fabrication of nano/microstructures for SERS substrates using an electrochemical method

**DOI:** 10.3762/bjnano.11.139

**Published:** 2020-10-16

**Authors:** Jingran Zhang, Tianqi Jia, Xiaoping Li, Junjie Yang, Zhengkai Li, Guangfeng Shi, Xinming Zhang, Zuobin Wang

**Affiliations:** 1 College of Mechanical and Electric Engineering, Changchun University of Science and Technology, Changchun, Jilin 130000, P.R. China; 2 Department of Pediatrics, The First Hospital of Jilin University, Changchun, Jilin 130021, P.R. China; 3 Institute of Advanced Wear & Corrosion Resistant and Functional Materials, Jinan University, Guangzhou 510632, P.R. China; 4 School of Mechanical Engineering, Shandong University of Technology, Zibo, Shandong, 255000, P.R. China

**Keywords:** electrochemical machining, gold (Au), lysozyme detection, magnesium (Mg), micro/nanopore, nano/microstructures, SERS substrate

## Abstract

Based on an electrochemical method, three-dimensional arrayed nanopore structures are machined onto a Mg surface. The structured Mg surface is coated with a thin gold (Au) film, which is used as a surface-enhanced Raman scattering (SERS) substrate. A rhodamine 6G (R6G) probe molecule is used as the detection agent for the SERS measurement. Different sizes of arrayed micro/nanostructures are fabricated by different treatment time using the electrochemical process. The topographies of these micro/nanostructures and the thickness of the Au film have an influence on the Raman intensity of the Mg substrate. Furthermore, when the thickness of Au film coating is held constant, the Raman intensity on the structured Mg substrates is about five times higher after a treatment time of 1 min when compared with other treatment times. The SERS enhancement factor ranges from 10^6^ to 1.75 × 10^7^ under these experimental conditions. Additionally, a 10^−6^ mol·L^−1^ solution of lysozyme was successfully detected using the Mg–Au nanopore substrates. Our low-cost method is reproducible, homogeneous, and suitable for the fabrication of SERS substrates.

## Introduction

Surface-enhanced Raman spectroscopy (SERS) can be used to detect biomolecules [1–3], explosives [4–6], and pesticide residues [7–9]. Plasmonic metal nanostructures are often used as SERS substrates to increase the molecule-specific Raman signal by several orders of magnitude. The functionality of SERS is due to a combination of surface electron movement in the substrate and charge transfer between substrate and the analyte molecules, in contrast to the typical signal intensity elicited during spontaneous Raman spectroscopy, which is extremely weak [10–11]. SERS is representative of other technologies that can amplify signal intensities based on strong electromagnetic fields and chemical enhancement [12–14].

Recently, all kinds of shapes of nanostructures machined by several researchers as SERS substrates have been machined by using lithography-based method [15–20]. Additionally, nanostructures are also fabricated by hybrid lithography [21–26] methods combined with dry etching or wet etching. For example, the commercial Klarite substrate [21–23] machined by electron beam lithography (EBL) and wet etching consists of 1 μm deep square-based pyramidal pits in the silicon surface. A rhodamine solution (10^−4^ mol·L^−1^) is then detected using the Klarite substrate. Candeloro et al. [24] employed EBL and reactive ion etching to machine nanoholes of 400 nm diameter and 50 nm depth. Subsequently, nanoholes were transferred onto the glass surface using the peeling template method and R6G molecules (10^−6^ mol·L^−1^) were used with the substrate for detection. Au nanostructures of different shapes and sizes (including grating, disk, and pyramid structures) have also been fabricated using EBL and reactive ion etching methods [25]. The Raman intensities of R6G and 4-mercaptopyridine molecules were measured by using different substrates. In addition, the Raman intensity of R6G on the pyramid structures was higher than that of R6G on the other structures in the experiment, and the enhancement factor of R6G molecules on the pyramid structure was about 10^5^. Wu et al. [26] machined nanohole array structures using EBL and lift-off methods. The diameter of the nanoholes ranged from 90 to 585 nm, and the gap between adjacent nanoholes ranged from 125 to 585 nm. An enhancement factor of 8 × 10^6^ was achieved for 4-mercaptobenzoic acid molecules on the arrayed Au nanoholes. However, lithography-based methods have limitations, as they are inefficient and cannot be exploited for mass production. In addition, it is challenging to use the existing methods to fabricate more complex nanostructures.

Focused ion beam (FIB) technology can also be used to directly fabricate high-precision nanostructures on surfaces made of silicon, silicon dioxide and metal [27–33]. FIB technology is therefore used as a processing method for SERS substrates. Using the FIB method, Lin et al. [29] fabricated micro/nanostructures on the surface of Au-coated single crystal silicon. By changing the etching time and current, micro/nanostructures with different size scales and geometric shapes (such as hexagons and pentagons) were obtained. Compared with other geometries, the hexagonal micro/nanostructure surface yielded the highest Raman intensity during the detection of R6G molecules. In addition, the spatial distance of the micro/nanostructures ranges from 22 to 83 nm, and the Raman intensity of R6G increases exponentially as the distance between adjacent micro/nanostructures decreases. Gao et al. [30] fabricated elliptical nanostructures and studied the effect of processing distance parameters and gold film thickness on the Raman intensity of R6G. They found that the Raman intensity of R6G was highest on densely packed structures. Additionally, the Raman intensity of R6G decreases as the number of hot spots decreases. Sivashanmugan et al. [32] employed FIB technology to prepare nanostructures on silicon surfaces, which were then coated with Au and Ag films to generate SERS substrates. The enhancement factor range of R6G using the substrate was between 2.62 × 10^6^ and 1.74 × 10^7^. Gao et al. [33] machined elliptic nanostructures with different parameters on Si substrates, including the spacing between structures and the thickness of the gold film. A spacing of 15 nm between the adjacent nanostructures was for the detection of R6G molecules with a concentration of 10^−6^ mol·L^−1^. Compared with other processing methods, the precision of FIB processing technology is relatively high. However, FIB processing is an expensive and low-throughput technology. In addition, the processing time of a wide range of micro/nanostructures is long. Therefore, the low-cost and efficient preparation of array nanostructures with controllable shape, size and density is urgently required for SERS substrates for molecular recognition.

Some researchers have fabricated nanostructures as SERS substrates by using electrochemical oxidation–reduction cycle (ORC) methods [34–39]. Generally, sheets of Au and Pt, and a KCl-saturated Ag/AgCl rod are used as the working, counter, and reference electrodes, respectively. Using this approach, Au/TiO_2_ nanocomposites formed on Pt substrates yielded a SERS enhancement factor of 1.8 × 10^8^ for R6G molecules [34]. Chang et al. [35] fabricated different Ag nanostructures on Pt substrates using a sonoelectrochemical ORC method with different ratios between the time periods of deposition and dissolution. The detection level of R6G molecules was 2 × 10^−13^ mol·L^−1^ and the highest enhancement factor achieved was 2.3 × 10^8^. Yang et al. [36] used ORC treatments in KCl solution to fabricate roughened Ag substrates. In this system, the limit of detection for R6G with SERS was 2 × 10^−8^ mol·L^−1^. Based on a ORC method, Chen et al. [37] created hybrid Au–AuO*_x_* with reverse rates of 200, 100, 50, 25, and 5 mV/s. The highest enhancement factor observed with R6G in this system was 5.5 × 10^6^ with a reverse rate of 25 mV/s. Furthermore, pigments of Brilliant Blue FCF and Indigo Carmine at concentrations as low as 10^−8^ mol·L^−1^ and 10^−7^ mol·L^−1^, respectively, were detectable using the SERS substrate. Ou et al. [38] prepared Ag SERS substrates by using triangular-wave ORC procedures in KCl solution. In this study, the Raman intensity of R6G (2 × 10^−6^ mol·L^−1^) on the Ag substrate was larger with subsequent drying treatment than without.

Anodic aluminum oxide (AAO) has been used as a mask to fabricate nanodot SERS substrates [40–43]. Using an AAO mask, Han et al. [41] manufactured graphene/Au nanodot array structures, which were used as SERS substrates. The diameter and gap distribution ranged from 30 to 42 nm and from 20 to 30 nm, respectively. In addition, a detection level of 10^−9^ mol·L^−1^ for R6G molecules was obtained using the aforementioned SERS substrates. Choi et al. [42] used a nanoporous template of AAO as a SERS substrate, and varied the thickness of either the Au film or the AAO itself. An enhancement factor of 10^7^ was obtained with an Au thickness of 20 nm and an AAO thickness of 100 nm. Using an AAO template, Aflatoxin B_1_ (AFB_1_) from peanut extract was detected at concentrations ranging from 1.5 µg/L to 1.5 mg/L. Although the method is suitable for the evaluation of AFB_1_ content in food safety inspections [43], the fabrication process for generating the AAO is lengthy.

In this paper, a simple and rapid electrochemical micromachining approach is presented for fabricating sensitive three-dimensional SERS substrates. First, by controlling the parameters of plasma electrolytic oxidation (PEO) treatment, arrayed nanopores were formed on a Mg surface. Then, the nanopore surfaces were coated by incubating them with Au films for different lengths of time. The nanostructures were fabricated by controlling the treatment time and R6G molecules were chosen to be adsorbed onto the substrate. Finally, the Raman intensities of low concentrations of lysozyme were determined using arrayed structures as the SERS substrates. Using this approach allows for the accurate quantification of extremely small amounts of protein.

## Experimental

**Figure 1:**
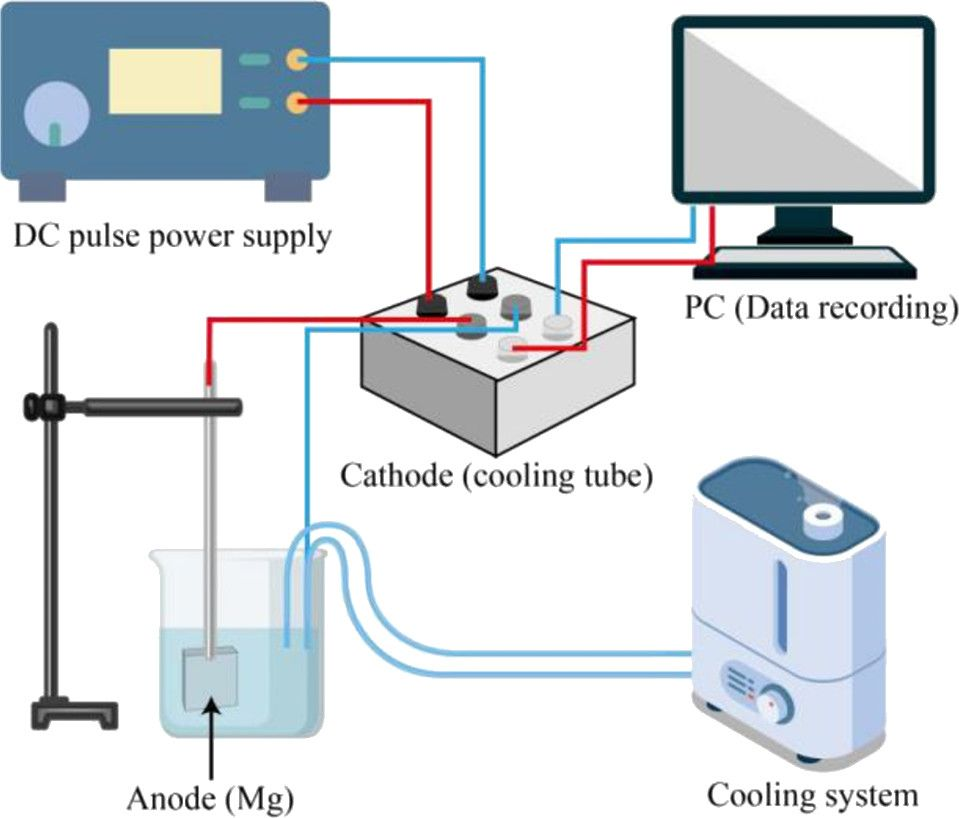
Schematic diagram of fabrication of the nanopores substrates using PEO processing.

As-cast Mg ingots were sliced into rectangular coupons (15 × 15 × 4 mm^3^) for anodic oxidation treatment. Prior to the treatment, all specimens were ground using SiC paper up to 1200 grit, and then degreased with ethanol and deionized water in succession. No further purification was carried out. A customized DC power supply was used to conduct the PEO. Figure 1 shows the schematic diagram of nanopore formation using PEO processing. The specimens and carbon tubes were utilized as the anode and cathode, respectively, and the electrolyte solution was prepared with 2 g/L KOH and 10 g/L Na_3_PO_4_ in deionized water. The PEO treatment was performed in constant-current mode with a fixed constant current density of 25 mA/cm^2^. The frequency and duty ratio were 500 Hz and 50%, respectively. The electrolyte temperature was regulated within 30 ± 2 °C by a mechanical stirring cooling system. To study the influence of surface conditions (e.g., porosity and roughness) on the SERS intensity, the duration of the PEO treatment was set as the single variable (1, 2, and 5 min), as shown in Table 1.

**Table 1:** Parameters used for nanopore fabrication on the Mg surface using the PEO method.

Condition	Parameter

Material	SiC-polished Mg ingot
Solution	2 g/L KOH and 10 g/L Na_3_PO_4_
Treatment method	Plasma electrolytic oxidation (PEO)
Electric current parameter	Constant current density 25 mA/cm^2^ Duty ratio 50% Frequency 500 Hz
Temperature	30 ± 2 °C
Preparation time	1 min 2 min 5 min

The structured Mg surfaces were coated with Au of varying thickness and dipped into an aqueous solution of R6G (10^−7^ mol·L^−1^) for 20 min. The excess R6G molecules were removed by rinsing with ethanol and a gentle nitrogen flow was used to dry the samples.

A micro-Raman inVia spectroscopic system (Renishaw, UK) with 532 and 785 nm lasers was used. The incident optical power was kept at 0.6 mW with a 50× objective and the beam diameter was approx. 1 μm. The signal detector used a CCD camera (1040 × 256 pixels) with a grating size of 1800 lines/mm. The exposure time was 1 s and one accumulation scan was employed. The mapping images of the Raman spectrum were scanned over a 20 × 20 μm^2^ area. Before the tests, the Raman spectra were rectified using a standard Si substrate. A Raman intensity peak of 1362 cm^−1^ for R6G was chosen in the experiment.

An atomic force microscopy (AFM) system (Dimension Icon, Bruker, Germany) was employed to detect the two-dimensional and three-dimensional topographies of the nanopores. Imaging was performed in contact mode and an elastic constant of 0.2 N/m was selected for the silicon cantilever. The scanning area was 50 × 50 μm^2^. In addition, a scanning electron microscopy (SEM) system (Zeiss, Germany) was employed to characterize the nanopores.

## Results and Discussion

### Fabrication of arrayed nanopores on the Mg surface

Surface roughness and chemical composition have a strong influence on the intensity of Raman signals. PEO was employed to fabricate a porous oxide layer on a Mg alloy surface, which benefits from the increase of surface roughness and shows the potential for storing micro- or nanoparticles. During PEO treatment, the intrinsic passivation layer of the Mg alloy is disrupted in random positions through local melting during electrical breakdowns. After cooling by the electrolyte, a stable oxide layer containing arrayed pores is deposited on the surface. The parameters and duration of the PEO process should be carefully determined, as intensive energy input and longer treatment duration may create excessive surface roughness or even introduce unexpected defects on the surface. Thus, a set of moderate parameters was applied in this work to ensure that the Raman signal reflection properties were optimal.

**Figure 2:**
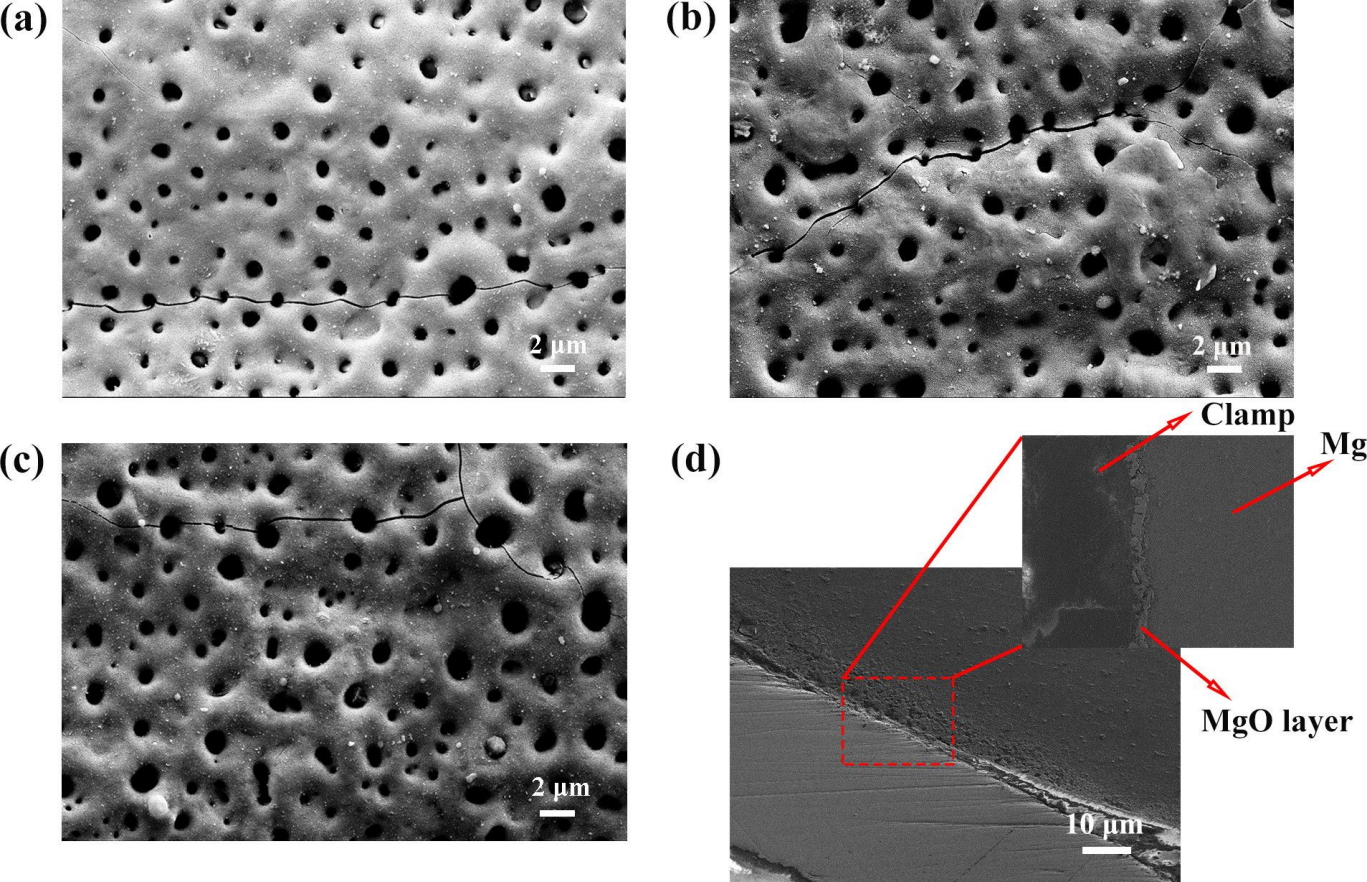
SEM images of the arrayed nanopores after different treatment times. SEM image of the arrayed nanopores after the treatment times of (a) 1 min, (b) 2 min, and (c) 5 min. (d) Cross-sectional SEM image of arrayed nanopores.

Figure 2 shows the two-dimensional topographies of the arrayed nanopores with different treatment time of 1, 2, and 5 min. Figure 2a shows the two-dimensional topographies of the arrayed nanopores after 1 min of treatment time. The depth and diameter of the nanopores gradually increased as the treatment time increased, as shown in Figure 2b,c. Figure 2d shows cross-sectional SEM images of arrayed nanopores. The surface morphology includes MgO and Mg layers. In addition, a clamp is used to fix the sample. As shown in Figure 2, all PEO-treated Mg specimens demonstrate the typical surface morphology, which comprises of micrometer and sub-micrometer-sized quasi-circular pores and cracks. This porous and uneven surface is a result of the consecutive dielectric breakdown of the passivation layer and the heat generated during that process. As a result, the average diameter of the pores increases as the duration of PEO duration increases.

**Figure 3:**
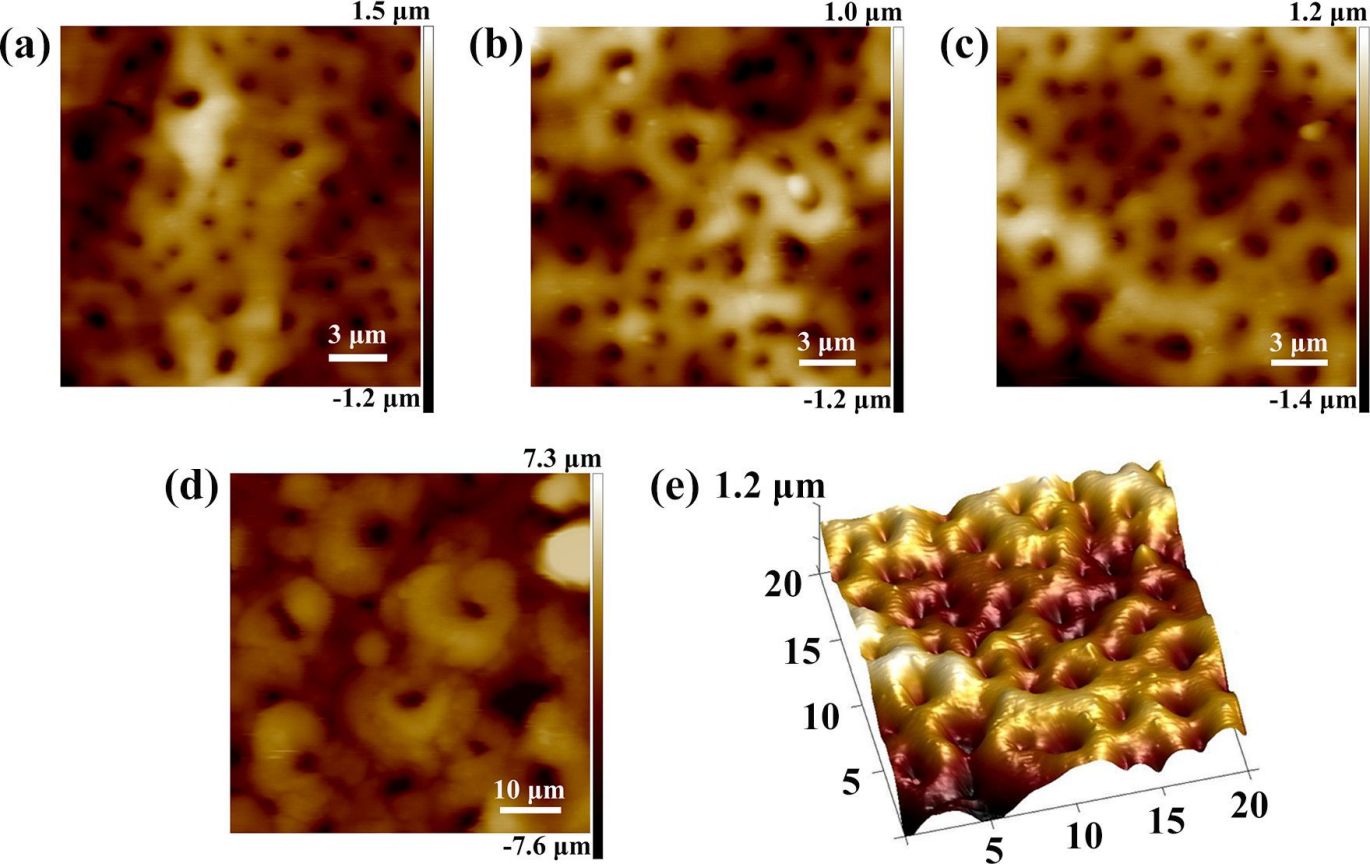
AFM images of the arrayed nanopores with different treatment time. AFM image of the arrayed nanopores after a treatment time of (a) 1 min, (b) 2 min, (c) 5 min, and (d) 10 min. (e) Three-dimensional AFM image of arrayed nanopores after a treatment time of 5 min (e).

Figure 3 shows AFM images of the arrayed nanopores after fabrication with different treatment times. After 1 min, the nanopore diameter and depth were 0.7 ± 0.25 µm and 0.5 ± 0.16 µm, respectively (Figure 3a). After 2 min, the nanopore diameter and depth were 0.9 ± 0.3 µm and 0.6 ± 0.15 µm (Figure 3b). When the treatment time was increased to 5 min, the nanopore diameter and depth were 1.5 ± 0.3 µm and 1 ± 0.1 µm, respectively, as shown in Figure 3c. When the treatment time was increased to 10 min, the nanopore diameter and depth were 7.2 ± 0.3 µm and 5 ± 0.5 µm, respectively, as shown in Figure 3d. Thus, a 10 min treatment time led to the formation of pores with microscale structure. A three-dimensional AFM image of arrayed nanopores after a treatment time of 5 min is shown in Figure 3e.

Structurally similar nanopores can be machined by using mechanical machining methods. For instance, micro/nanostructures can be fabricated using diamond turning, such as single point diamond turning or diamond fly cutting. For example, sinusoidal grid surfaces can be formed with the aid of fast tool servos. Nanopores with depths of 6.33 µm can be generated by using diamond turning [44].

### Raman intensity of R6G molecules on the arrayed nanopores

The PEO treatment time and the thickness of the Au film can have a significant impact on the performance of the SERS substrate. The performance was quantified by measuring Raman enhancement, which was determined by using R6G as a probe molecule.

#### Raman intensity with different thicknesses of the gold film

The effect of Raman enhancement of R6G molecules with different thicknesses of the gold film on the same structure surface (10, 20, and 30 nm) was studied. The data of Raman mapping were exported from the Raman spectra point by point.

**Figure 4:**
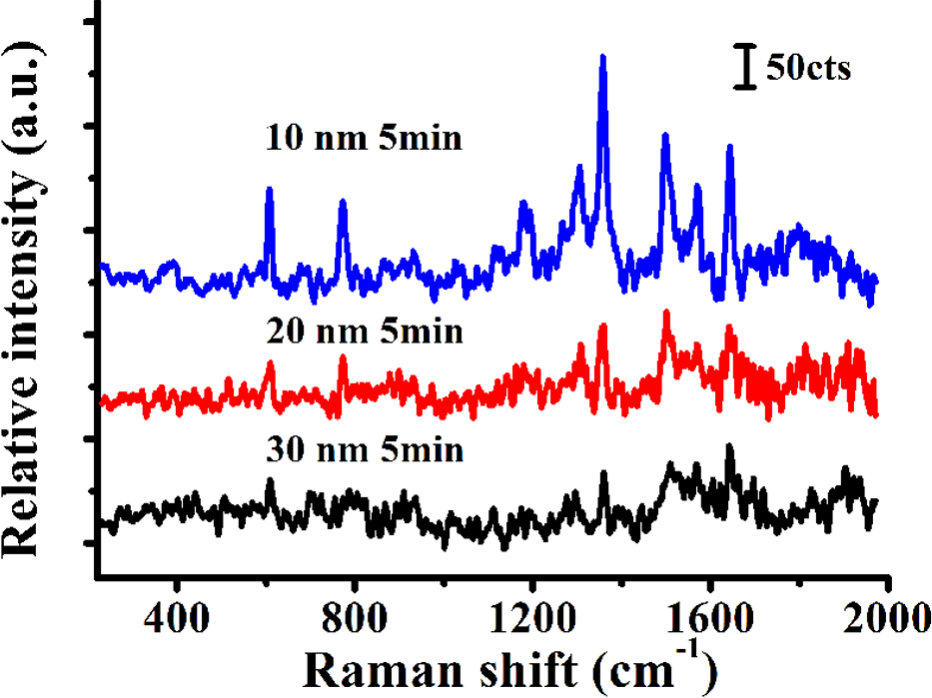
Raman spectra of R6G molecules (10^−7^ M) measured using Au-coated nanopore substrates with Au film thicknesses of 10, 20, and 30 nm.

Figure 4 shows the Raman spectra of R6G molecules (10^−7^ M) on nanopore substrates coated with different thicknesses of the Au film after a constant time of PEO treatment (5 min). The characteristic Raman peaks of R6G molecules were detected at 611, 772, 1183, 1311, 1362, 1503, and 1605 cm^−1^. The results indicate that the Raman signal was most intense when the nanopore substrate with a 10 nm thick Au film was used. The Raman intensity of R6G decreased as the thickness of the Au film increased. Accordingly, the Raman intensity of the R6G signal derived from substrates coated with a 30 nm thick Au film was very low, as shown in Figure 4. Overall, the results show that the Raman intensity of R6G is affected by the thickness of the Au film. The effect of Au thickness on the electric field intensity has previously been studied [45–47]. Zhang et al. [45] used a self-assembled method to fabricate PS nanosphere array substrates with Ag films of different thickness. The strongest electric field intensity enhancement was generated with a 10 nm thick Ag film. Using the AFM-based scratching method, Wang et al. [46] obtained nanodot array structures fabricated with Au films of different thickness. The Au were 13, 20, and 40 nm thick. The results show that a 13 nm thick Au film conferred the best enhancement effect. Therefore, the use of a thinner Au film can improve the Raman intensity of probe molecules. Cao et al. [47] employed femtosecond laser irradiation to fabricate nanorod arrayed structures decorated with Au nanoparticles. The study showed that the Raman intensity tended to decrease as the Au film thickness increased. Based on the above results, we selected Au films of 10 nm thickness for further quantification of the Raman intensity of probe molecules.

#### Raman intensity after different treatment times

**Figure 5:**
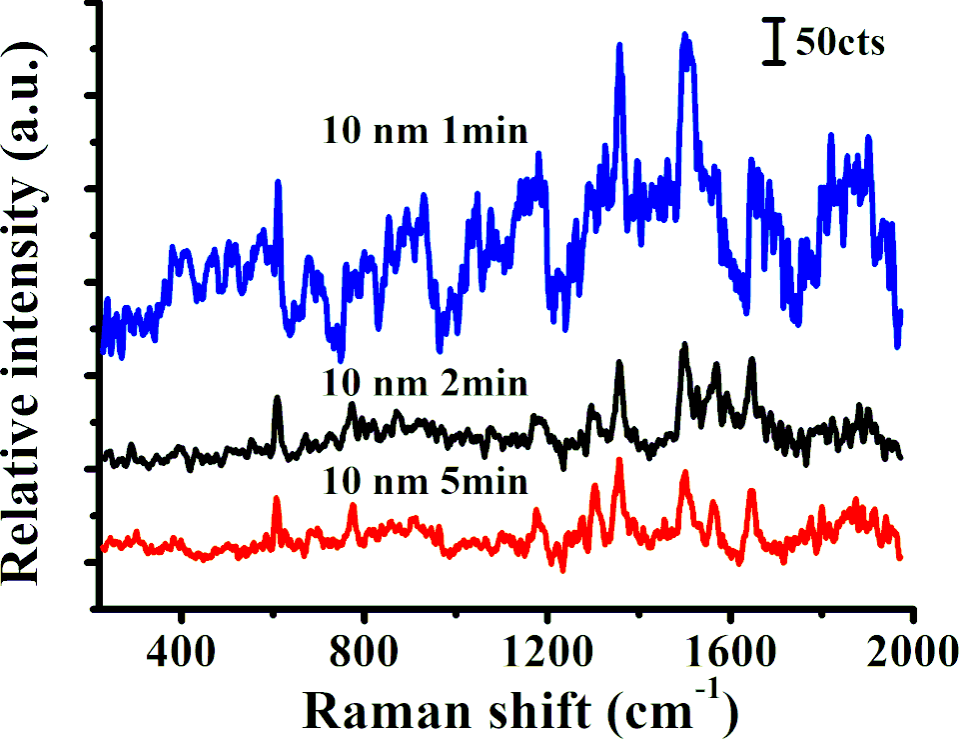
Raman spectra of R6G molecules (10^−7^ M) after different PEO treatment times (1, 2, and 5 min).

Figure 5 shows the Raman spectra of R6G molecules (10^−7^ mol·L^−1^) on nanopore structures that were fabricated using PEO different treatment times. The microstructures vary in their morphology depending to the length of the treatment time, which in turn has a profound effect on the Raman intensity. Compared with other structures generated using different treatment times, a bare surface did not yield a high Raman intensity when measuring R6G. The Raman intensity was strongest when surfaces were PEO-treated for 1 min and a Au film of 10 nm thickness was deposited (Figure 5 and Figure 2a). Under these conditions nanopores with smaller dimensions were formed than after treatment times of 2 and 5 min. The Raman intensity of R6G molecules was three-fold higher after a treatment time of 1 min than after treatment times of 2 and 5 min.

A single-cavity structure can significantly enhance the Raman signal [48–50]. Chang et al. [48] fabricated cavities by using an indentation method and found that the Raman intensities of R6G were influenced by indentation depth and tip-to-tip displacement. In our previous studies [49–50], a cavity depth of 1.7 µm was generated using a normal force of 10 mN with the force modulation indentation method. However, nanocavities were formed by the overlap of adjacent cavities. The depth of the nanocavities reached ca. 200 nm as the machining feeds were reduced. In addition, the Raman intensity reported by the R6G probe on the nanostructures was ten times that of a single-cavity structure.

**Figure 6:**
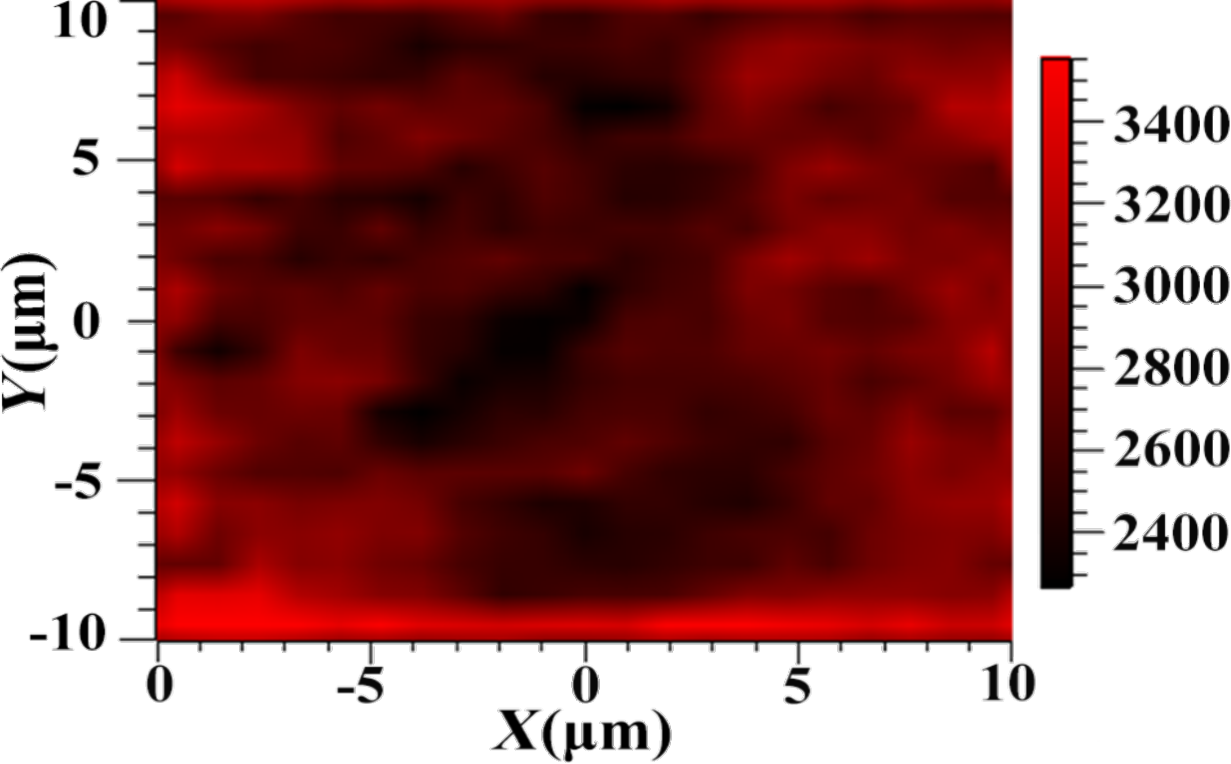
Raman intensity mapping image of arrayed nanopores formed after a treatment time of 2 min with a Au coating of 10 nm thickness.

Figure 6 shows the Raman intensity mapping image of arrayed nanopores formed after a treatment time of 2 min with a Au coating of 10 nm thickness. The Raman signal of R6G molecules could be detected. Thus, the electrochemical method can be used to create flexible Au-coated substrates of highly reproducible structure with long-term stability.

#### SERS measurement of lysozyme on the nanopore substrates

The enzyme lysozyme can rupture the cell walls of certain pathogens following activation of the innate immune system [51]. However, excess lysozyme activity can increase the incidence of some diseases. In certain kinds of cancer, such as leukemia [52], excessive production of lysozyme is toxic and can induce organ disorder.

**Figure 7:**
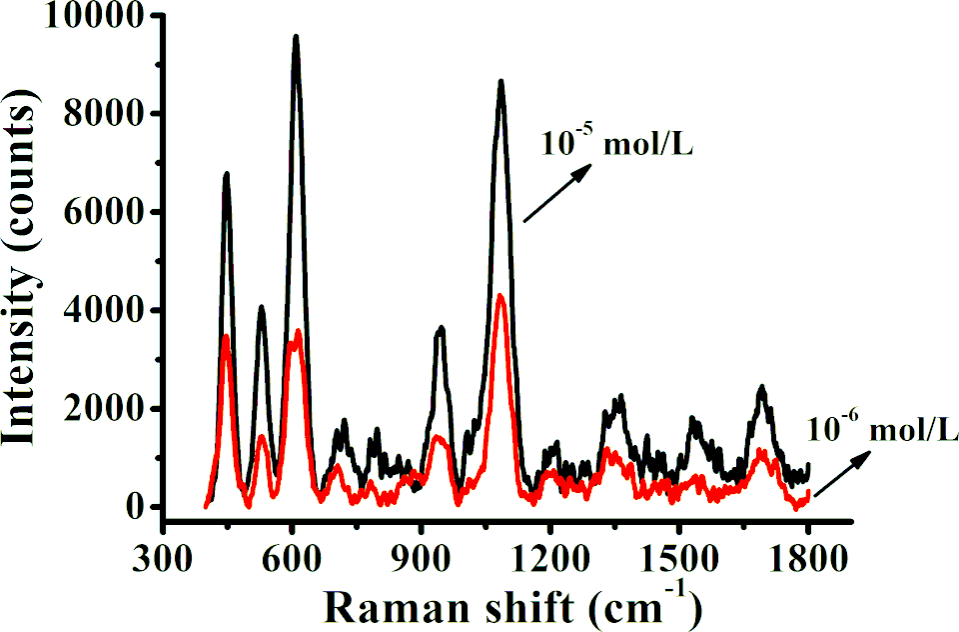
Raman spectra of lysozyme molecules of 10^−6^ mol·L^−1^ and 10^−5^ mol·L^−1^ on nanopore substrates fabricated with a treatment time of 1 min and coated with a 10 nm Au film.

Figure 7 shows the SERS spectra of lysozyme (10^−6^ mol·L^−1^ and 10^−5^ mol·L^−1^) in ethanol solution on nanopore substrates that were fabricated with a PEO treatment time of 1 min and were coated with a Au film of 10 nm thickness. The characteristic Raman peaks of lysozyme molecules were detected at concentrations as low as 10^−6^ mol·L^−1^. These peaks included SS bridge (521 cm^−1^), phenylalanine (601 cm^−1^), tryptophan(s) (760 cm^−1^), tyrosine doublet (858 cm^−1^), tryptophan (881 cm^−1^), CC stretching (934 cm^−1^), tyrosine (1085 cm^−1^, 1210 cm^−1^), tryptophan (1337 cm^−1^), COO− symmetric stretch(s) (1384 cm^−1^), and tryptophan (1554 cm^−1^). Together, these data show that the electrochemical method can be used to fabricate nanopores as SERS substrates for the sensitive detection of proteins such as lysozyme.

## Conclusion

Two- and three-dimensional arrayed micro/nanopores can be machined on Mg substrates by using a novel electrochemical method. The optimal treatment time for the process was 1 min, and the SERS intensity of the R6G molecules was enhanced by applying a 10 nm Au film onto the structured Mg surface. The SERS enhancement factor of this optimized system was between 10^6^ and 1.75 × 10^7^. Experiments with lysozyme demonstrated that the Mg–Au nanopore substrates can be used to detect low levels of proteins (10^−6^ mol·L^−1^). Due to its reliability, homogeneity, low cost and high sensitivity, the system described herein holds great promise for future protein detection and quantification applications.
